# sc-PDB: a 3D-database of ligandable binding sites—10 years on

**DOI:** 10.1093/nar/gku928

**Published:** 2014-10-09

**Authors:** Jérémy Desaphy, Guillaume Bret, Didier Rognan, Esther Kellenberger

**Affiliations:** Laboratoire d'innovation thérapeutique, Medalis Drug Discovery Center, UMR7200 CNRS-Université de Strasbourg, F-67400 Illkirch, France

## Abstract

The sc-PDB database (available at http://bioinfo-pharma.u-strasbg.fr/scPDB/) is a comprehensive and up-to-date selection of ligandable binding sites of the Protein Data Bank. Sites are defined from complexes between a protein and a pharmacological ligand. The database provides the all-atom description of the protein, its ligand, their binding site and their binding mode. Currently, the sc-PDB archive registers 9283 binding sites from 3678 unique proteins and 5608 unique ligands. The sc-PDB database was publicly launched in 2004 with the aim of providing structure files suitable for computational approaches to drug design, such as docking. During the last 10 years we have improved and standardized the processes for (i) identifying binding sites, (ii) correcting structures, (iii) annotating protein function and ligand properties and (iv) characterizing their binding mode. This paper presents the latest enhancements in the database, specifically pertaining to the representation of molecular interaction and to the similarity between ligand/protein binding patterns. The new website puts emphasis in pictorial analysis of data.

## INTRODUCTION

The 3D structures of macromolecules, as collected by the Worldwide Protein Data Bank (PDB) organization (http://wwpdb.org, (1)), offer wealth of information for computer-aided approaches to drug design. During the last 30 years, the steady increase of the PDB archive (2) has prompted the development of 3D methods for hit identification by virtual screening of chemical libraries, *de novo* ligand design and hit to lead. Many success stories have been reported in the literature (3). Besides, some proteins have never been efficiently modulated by chemical compounds despite intense efforts in medicinal chemistry. The concept of ligandability has thus been suggested to qualify the ability of a protein to bind with high affinity a small molecular weight compound (4,5). Recent studies demonstrated that simple geometric and physico-chemical descriptors of protein cavities (principally size, shape and polarity) are sufficient to predict structural ligandability (6–9).

**Figure 1.**
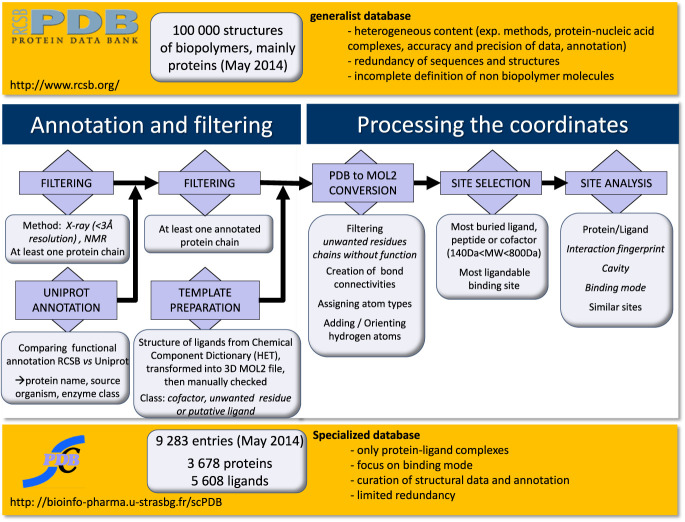
The general flow chart from PDB to sc-PDB.

The sc-PDB is a specialized structure database focused on ligand binding site in ligandable proteins (10). We have selected in the PDB all proteins in complex with a small synthetic or natural ligand (140 Da < MW < 800 Da), provided this ligand was well buried and biologically relevant and since 2013 provided the binding site was predicted ligandable according to a machine leaning-based model. The different stages of database design process are detailed in the online documentation and summarized in Figure 1.

The first publicly available version of sc-PDB has been released in 2004. The database has been annually updated with regular additions of new features (See Supplementary Table S1 for a summary of changes since the database creation). Not only the quality and the precision of data improved over the 10 years, but new tools have allowed global analysis of data. A major example is the clustering of sites for proteins present in multiple copies in the database (11). The new functionalities in sc-PDB, introduced after 2011, are discussed in detail in this paper.

## sc-PDB CONTENT

The sc-PDB data are directly compatible with computational methods, such as docking, molecular mechanics and electrostatic calculations. Unlike the PDB, which generally does not represent hydrogen atoms nor defines ionization state of titratable groups, the sc-PDB provides an all-atom model of molecules: (i) hydrogen atoms are added to amino acids considering that arginine and lysine are positively charged and aspartic and glutamic acids are negatively charged, (ii) hydrogen atoms are added to other residues according to ionized templates built from HET group dictionary (12), (iii) the intermolecular H-bonding network is optimized using the BioSolveIT Hydescorer program (13). The overall processing of an original PDB entry yields atomic data for a single ligand, the protein chain(s) surrounding this ligand and its binding site (i.e. all protein residues with at least one heavy atom closer than 6.5 Å to any ligand heavy atom). Of note, protein and binding site contain standard amino acids, and may include cofactor(s), metallic ion(s) and covalently bound residue(s), such as carbohydrate. Last, each sc-PDB entry is characterized with functional and chemical annotations.

The current sc-PDB release contains 9283 entries, representing 3678 different UniProt (14) proteins and 5608 different HET ligands. The data set is non-redundant: although about 10% of ligands and almost half of proteins are present more than once in the database, each sc-PDB ligand/protein complex is unique. Less than 5% of proteins are encountered more than 10 times in the database, yet some of them have a very high copy number. The three most frequent proteins are HIV protease (248 entries), cyclin-dependent kinase 2 (180 entries) and beta-secretase 1 (155 entries). More statistics are given in Supplementary Figure S1, and at http://cheminfo.u-strasbg.fr/scPDB/ABOUT.

The total size of compressed database is 1.5 GB. Its downloadable content is summarized in Table 1.

**Table 1.** Downloadable content of sc-PDB

filename	Number of entries in file	Data description	Data type
protein.mol2	one or list of PDB ID matching search criteria	All-atom description of sc-PDB protein(s)	Atomic data
ligand.mol2	one or list of PDB ID matching search criteria	All-atom description of sc-PDB ligand	Atomic data
site.mol2	one or list of PDB ID matching search criteria	All-atom description of sc-PDB ligand binding site	Atomic data
cavity6.mol2	one or list of PDB ID matching search criteria	The cavity is the negative image of the binding site, described by regularly spaced points colored according to pharmacophoric properties of the site atoms	Atomic data
ints_M.mol2	one or list of PDB ID matching search criteria	Non-bonded interactions between sc-PDB ligand and its binding site	Atomic data
		Each interaction is characterized by three points, placed respectively on the protein atom in interaction, the ligand atom in interaction and at the center of the segment defined by these two points	
ifp.txt	one or list of PDB ID matching search criteria	Non-bonded interactions between sc-PDB ligand and its binding site	Binary string
		For each residue in site is marked the presence or absence of interaction with ligand (hydrophobic contact, aromatic bond, H-bond, ionic bond, metal-ion bond)	
C-*clusterID*.tar.gz	Clusters of binding sites	The archive classifies all the sc-PDB entries of a UNIPROT protein. It is organized into directories, one for each cluster of sites. Each cluster contains protein.mol2, site.mol2 and ligand.mol2 files of all individual PDB entries, which have been 3D-aligned to the site at cluster center	Atomic data
Alignment.tar.gz	Pair of PDB ID, 3D-aligned for optimizing site similarity (1) or binding mode similarity (2)	The archive describes the protein, site, ligand and cavity (1) or non-bonded interaction (2) files for the reference entry (original coordinates) and the compared entry (fitted coordinates)	Atomic data
scPDB_results	list of entries matching search criteria	Annotation and 2D structure of ligands in csv, xlsx or sdf formats	Text
*PDBID*_distribution.tsv	list of entries similar to query site (1) or query binding mode (2)	Similarity scores	Text

All charts and pictures of sc-PDB website are downloadable in png format. The complete database is downloadable as a compressed archive from the database homepage.

## NEW FEATURES OF THE sc-PDB DATABASE

### Depiction of protein–ligand complexes

The latest sc-PDB release enables the user to depict protein–ligand complexes according to different needs and complexity levels. For example, a medicinal chemist may be primarily interested in the PoseView 2D sketch highlighting the ligand structure, binding site boundaries and main interactions (Supplementary Figure S2A). A cheminformatician may focus on the nearby tabulated list of protein–ligand interactions including involved atoms and a full topological description (distance, angle) of each interaction (Supplementary Figure S2B). Last, a structural biologist can access a 3D picture of the complex embedded in the OpenAstex viewer (Supplementary Figure S2B) (15). The interaction table is graphically linked to the 3D picture: scrolling the mouse over any interaction line in the table interactively displays the corresponding interaction in the neighboring 3D picture.

### Water molecules

Water is by essence the biological fluid. The role of water in molecular recognition events is not yet fully understood although it has been extensively studied experimentally and theoretically (see (16) for a comprehensive review). Observations made for water molecules at binding interface between a drug and its protein target demonstrated that ordered solvent molecule(s) can either reinforce or by contrast weaken the stability of the complex depending on the studied system (17). In drug design, interfacial water molecules have a profound impact on calculations, both the inexpensive computational protocols, such as hit finding by high-throughput docking (18), and the more sophisticated algorithms, such as lead optimization using free-energy perturbation calculations (19).

**Figure 2.**
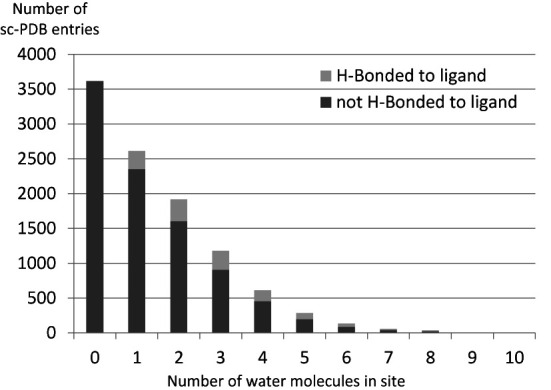
Crystallographic water molecules in sc-PDB binding sites.

Since 2012, a sc-PDB protein contains all water molecules that establish two or more hydrogen bonds with the binding site (i.e. donor-acceptor distance < 3.5 Å and 120° < donor-H-acceptor angle < 240°). These water molecules are expected to be hardly displaceable by a ligand because of tight binding to the protein (20). Water molecules are present in about two-thirds of sc-PDB complexes (Figure 2). The number of water molecules per site ranges from 1 to 10, but the distribution is largely biased toward smaller values. Although only few of the selected water molecules are in direct interaction with the ligand, using this information is key to structure-based design and drastically influences virtual screening for example.

### Query for similar binding sites

The molecular basis of the ligand/protein recognition gives insights into the specificity of a drug for its target protein. For example, structural variations in binding site may explain the permissive binding of different ligands to a single protein. As mentioned in the Introduction, we have previously addressed this issue by analyzing the multiple binding sites in a given protein (11). The sc-PDB clusters of binding sites can reveal differences in location, size, composition or 3D structure. For example, clustering the sc-PDB sites of adenylosuccinate synthetase yields three clusters; two of them that have similar structures and compositions except guanosine diphosphate (GDP) and Mg^2+^ cofactors; the third one is localized in a different region in the protein (Supplementary Figure S3). Other high quality databases derived from the PDB also facilitate the comparison of the binding sites across a protein family (21–23). The sc-PDB database is, however, the only meta-database enabling to search the PDB using user-defined queries mixing protein, ligand, binding site and binding mode properties. For example, a single query in the sc-PDB enables the selection of all protein–ligand complexes for which (i) the target is a protein kinase, (ii) the ligand is a fragment with a molecular weight between 150 and 300, (iii) the binding site comprises at least one bound water molecule, (iv) the ligand is neutral and contacts its target by one aromatic face-to-face interaction.

Local structural similarity between non-homologous proteins can account for the promiscuity of a ligand, and thus can help explaining the side effects of a drug or suggest its repositioning toward a novel target and therapeutical indication (24,25). The sc-PDB database now enables the identification of similar sites in distinct proteins using a pre-computed all-against-all comparison with the in-house developed Shaper algorithm (8). The sc-PDB website allows to query the matrix of scores for any given sc-PDB site. It displays the distribution of scores and lists the entries whose similarity score is higher than a given threshold (default value is 0.44). For example, the binding site for phosphomethylphosphonic acid-guanylate ester in *Escherichia coli* adenylosuccinate synthetase (PDB ID: 1HOP, HET: CGP) shares significant 3D similarity with a single site in sc-PDB, that of GTP in a murine homologous protein (Supplementary Figure S4).

### Query for similar binding patterns

The non-bonded interactions between a ligand and its protein define a 3D pattern that characterizes the binding mode. We have recently developed a new geometrical method to encode and compare protein–ligand interaction patterns (26). Briefly, each interaction is represented by three points: the interacting ligand atom, the interacting protein atom and a pseudo-atom at the geometric center of the above-cited two atoms. Each interaction is assigned a molecular type according to the type of non-bonded interaction (hydrophobic, aromatic, hydrogen bond, ionic bond, metal-ion bond). The 3D pattern is defined by all the triplets of interaction pseudo-atoms and graph theory is applied to find the maximal common subgraph (clique) between two 3D patterns. The similarity score evaluates the quality of overlap after 3D alignment of the two patterns. Using this approach we recently demonstrated that the protein–ligand binding mode is generally conserved within a family of homologous protein even though bound ligands are dissimilar.

**Figure 3.**
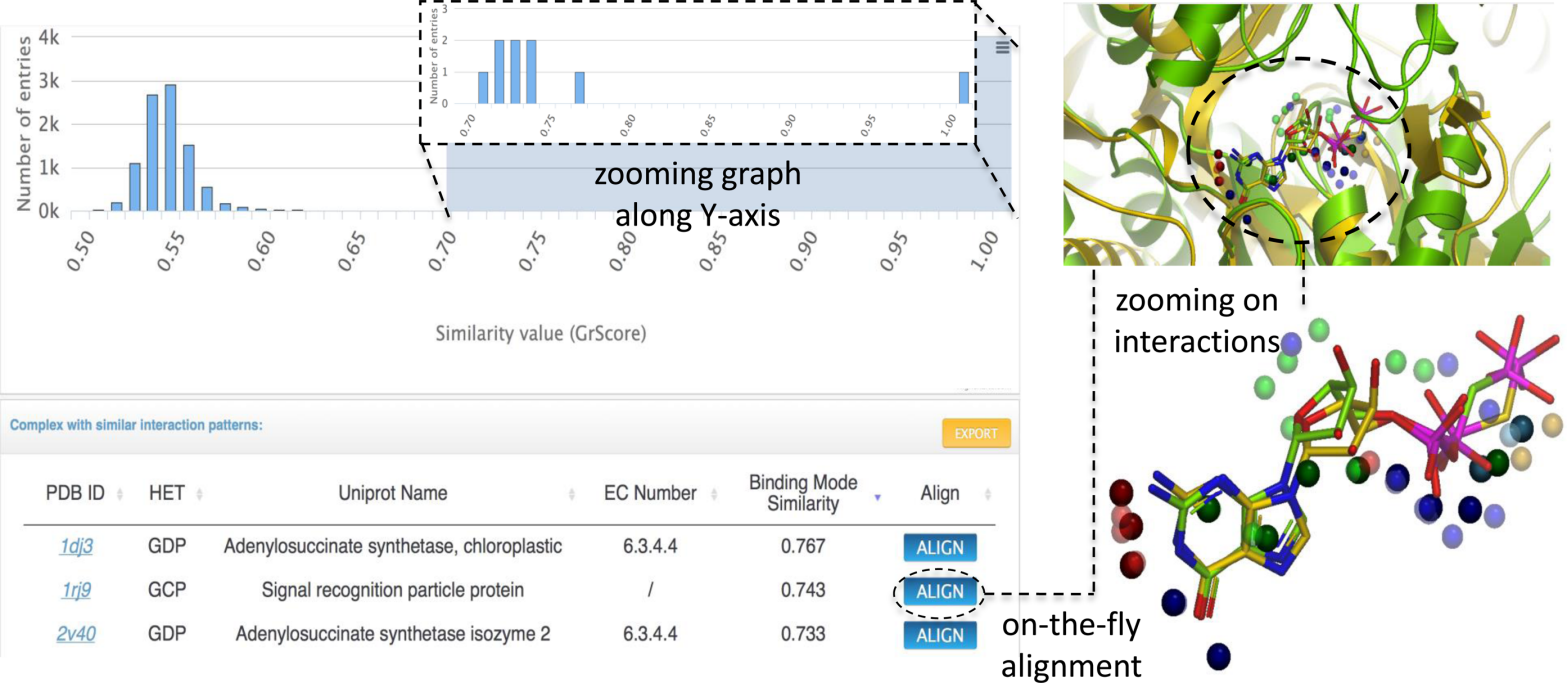
Search the sc-PDB for similar binding modes. Screenshots display the distribution of values for a given query binding mode (top left), the ranked list of similar entries (bottom left) and the 3D alignment of a selected hit with the query complex (top right). The closer view (bottom right) better shows aligned interaction points. The 3D structure of the query is colored in yellow (PDB ID: 1hop, HET: GCP), the selected hit in green (PDB ID: 1rj9, HET: GCP). Interaction pseudo-atoms are colored by interaction type (green, hydrophobic; blue, H-bond with ligand acceptor; red, H-bond with ligand donor; brown, metal chelation).

The sc-PDB database now enables the identification of similar 3D pattern in distinct complexes; the all-against-all comparison of sc-PDB complexes was computed using the program Grim (26). The sc-PDB website allows to query the matrix of scores for any given sc-PDB ligand/protein binding mode. It displays the distribution of scores and lists the entries whose similarity score is higher than the threshold selected on the distribution (default value is 0.65). For example, the binding mode of phosphomethylphosphonic acid-guanylate ester to *E. coli* adenylosuccinate synthetase (PDB ID: 1HOP, HET: CGP) shares significant similarity with 25 complexes in sc-PDB, representing 19 different proteins bound to GDP, GTP or close analogs. The two top scorers are respectively a homologous protein in wheat (PDB ID: 1DJ3) and the functionally unrelated signal recognition particle protein (PDB ID: 1RJ9, Figure 3).

### A new interface

The main architecture of database has not been changed, but the sc-PDB website has been completely re-designed to enhance interactivity. For every entry, the user can navigate in the main menu and directly switch views in the same window focusing on either a simple description of the entry, or a full characterization of the ligand or its binding site. Only searches for similar binding sites or binding modes open a new window with the rank-ordered list of sc-PDB hits corresponding to the query. At almost all sections of the web interface, molecules (protein, ligand, binding site), interaction pattern, tabulated results (hit lists, protein–ligand interactions) and charts (ligand and binding site properties, distribution of similar binding sites or binding modes) can be downloaded in the relevant file format (mol2, xlsx, csv, tsv, png, jpg, svg, pdf). In case of binding site/binding mode similarity searches, aligned molecules are also downloadable (Table 1).

The website specifications are detailed at http://cheminfo.u-strasbg.fr/scPDB/ABOUT.

## SUPPLEMENTARY DATA

Supplementary Data are available at NAR Online.
